# Neurotherapy of Yi-Gan-San, a Traditional Herbal Medicine, in an Alzheimer’s Disease Model of *Drosophila melanogaster* by Alleviating Aβ_42_ Expression

**DOI:** 10.3390/plants11040572

**Published:** 2022-02-21

**Authors:** Ming-Tsan Su, Yong-Sin Jheng, Chen-Wen Lu, Wen-Jhen Wu, Shieh-Yueh Yang, Wu-Chang Chuang, Ming-Chung Lee, Chung-Hsin Wu

**Affiliations:** 1 School of Life Science, National Taiwan Normal University, Taipei 11677, Taiwan; mtsu@ntnu.edu.tw (M.-T.S.); zxx159753@gmail.com (Y.-S.J.); kumo.lu@gmail.com (C.-W.L.); efgy78@gmail.com (W.-J.W.); 2 MagQu Co., Ltd., New Taipei City 11231, Taiwan; syyang@magqu.com; 3 Sun Ten Pharmaceutical Co., Ltd., Taipei 23143, Taiwan; cwctd331@sunten.com.tw; 4 Brion Research Institute of Taiwan, Taipei 23143, Taiwan; mileslee@sunten.com.tw

**Keywords:** Alzheimer’s disease, amyloid β, immunomagnetic reduction, *Drosophila melanogaster*

## Abstract

Alzheimer’s disease (AD), a main cause of dementia, is the most common neurodegenerative disease that is related to the abnormal accumulation of amyloid β (Aβ) proteins. Yi-Gan-San (YGS), a traditional herbal medicine, has been used for the management of neurodegenerative disorders and for the treatment of neurosis, insomnia and dementia. The aim of this study was to examine antioxidant capacity and cytotoxicity of YGS treatment by using 2,2-Diphenyl-1-picrylhydrazyl (DPPH) and 3-(4,5-Dimethylthiazol-2-yl)-2,5-diphenyltetrazolium bromide (MTT) assays in vitro. We explored neuroprotective effects of YGS treatment in alleviating Aβ neurotoxicity of Drosophila melanogaster in vivo by comparing survival rate, climbing index, and Aβ expressions through retinal green fluorescent protein (GFP) expression, highly sensitive immunomagnetic reduction (IMR) and Western blotting assays. In the in vitro study, our results showed that scavenging activities of free radical and SH-SY5Y nerve cell viability were increased significantly (*p* < 0.01–0.05). In the in vivo study, Aβ_42_-expressing flies (Aβ_42_-GFP flies) and their WT flies (mCD8-GFP flies) were used as an animal model to examine the neurotherapeutic effects of YGS treatment. Our results showed that, in comparison with those Aβ_42_ flies under sham treatments, Aβ_42_ flies under YGS treatments showed a greater survival rate, better climbing speed, and lower Aβ_42_ aggregation in *Drosophila* brain tissue (*p* < 0.01). Our findings suggest that YGS should have a beneficial alternative therapy for AD and dementia via alleviating Aβ neurotoxicity in the brain tissue.

## 1. Introduction

Dementia is the most frequent age-related neurocognitive disorder. Patients with dementia are known to frequently experience disturbing behavioral and psychological symptoms, such as excitement, aggression, hallucinations, insomnia, anxiety, wandering, and depression [1,2,3]. Alzheimer’s disease (AD), a main cause of dementia, is the most common neurodegenerative disease that is related to the abnormal accumulation of amyloid β (Aβ) proteins [4]. Pathological indicators of AD include the presence of Aβ plaques, which damage neurons, particularly those surrounding the hippocampus [5]. Aβ plaques are neuropathological biomarkers for AD. The challenge with assaying AD biomarkers is ascribed to the ultralow concentrations of Aβ_42_ proteins in the cerebrospinal fluid and the blood [6].

Yi-Gan-San (YGS, Shun-Ning-Yi OTC medicine), a traditional Chinese (Kampo) herbal medicine, is composed of Atractylodes, Poria, Chuanxiong, Angelica, Bupleurum, Licorice and Uncaria. Since ancient times, YGS has been used to treat patients who have symptoms, such as nervousness, short temper, irritability and sleeplessness. Recently, several studies have shown that the administration of YGS is effective for treating the behavioral and psychological symptoms of dementia (BPSD) [7,8]. In Japan, YGS is also called yokukansan and served as a remedy for restlessness and agitation in children [9]. Yokukansan has been approved by the Japanese Ministry of Health, Labor and Welfare as a remedy for neurosis, insomnia, irritability and night crying in children. YGS is the extract of multiple crude drugs containing a large number of ingredients. YGS can protect against the cytotoxic effect of a low concentration of corticosterone on hippocampal neurons [10]. To date, over 70 basic research articles have been published on the pharmacological efficacy and mechanisms of YGS about the pharmacokinetics, metabolism and brain distribution of their active ingredients. Among these research articles, YGS has been confirmed as a new potential therapeutic agent for the management of neurodegenerative disorders such as AD, and for the treatment of neurosis, insomnia and dementia [7,8,11,12,13,14]. Although molecular complexity is the main obstacle to studying the mechanism of YGS, it is an advantage for various pharmacological effects.

The argument that recapitulation of human AD by using transgenic animal models has improved the understanding of its pathological mechanisms is indisputable. The *Drosophila melanogaster* model has made its mark as an effective tool for the study of human AD [15,16,17], including its cellular aspects and associated physiological and behavioral traits, through the use of both conventional and innovative genetic tools. It is undeniable that human genetics research has improved the understanding of genes related to neurodegenerative diseases. However, the inspection of human subjects is hampered by moral and technical limitations. Therefore, many studies turn to AD animal models, such as the fruit fly (*Drosophila melanogaster*), mouse (*Mus musculus*), zebrafish (*Danio rerio*), and nematode (*Caenorhabditis elegans*); with each mirroring differing aspects of AD to generalize human diseases. Among these animal models, the *Drosophila melanogaster* AD model has been selected as an ideal tool to study AD disorders because targeted expression of Aβ proteins in adult *Drosophila* can result in changes in the appearance of the structure, including a reduction in external eye size, and a loss of ommatidia organization. Thus, this study selected *Drosophila* as an AD model as a drug discovery tool for AD. Pathological evidence of AD includes the presence of Aβ protein in the brain tissue [18,19,20]. It is very important to find suitable analytical methods that can detect Aβ expression in *Drosophila* brain tissue. The novel techniques that have been successfully developed to detect Aβ in cerebrospinal fluid (CSF) are clearly suitable for detecting Aβ expression in *Drosophila* brain tissue [21,22]. For example, immunomagnetic reduction (IMR) can detect ultralow concentrations of Aβ protein in human CSF and blood for early diagnosis of AD through the use of antibody-functionalized magnetic nanoparticles dispersed in an aqueous solution [23,24,25,26].

In this study, we shed light on how *Drosophila melanogaster* became an animal model for AD, as well as its contribution as a tool for discovering therapeutic drugs for AD. We used highly sensitive IMR assay technology that is capable of detecting ultralow concentrations of Aβ in *Drosophila* brain tissue. Our results demonstrated that YGS treatments had better antioxidant capacity, and low cytotoxicity for SH-SY5Y nerve cells for the in vitro study, and had a greater survival rate, better climbing speed, and lower Aβ_42_ aggregation in the brain tissue. On the basis of our data, YGS treatment may be a beneficial therapy for alleviating neurodegenerative disorders in AD.

## 2. Results

### 2.1. Chromatographic Fingerprints of YGS

**Figure 1:**
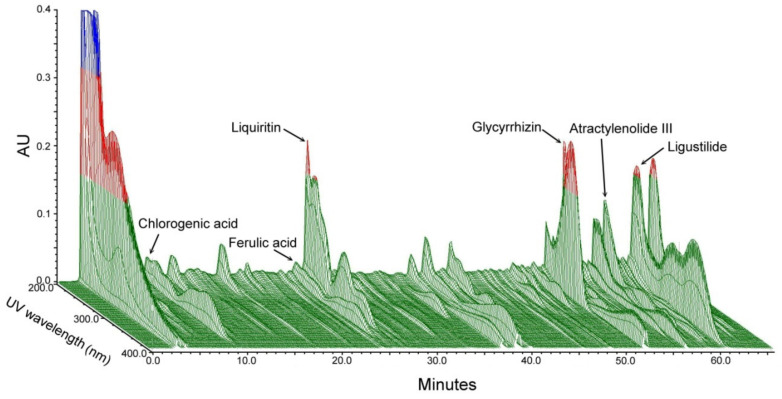
Chromatographic fingerprints of YGS from 3D HPLC. The bioactive marker compounds, namely chlorogenic acid, ferulic acid, liquiritin, glycyrrhizin, atractylenolide III, and ligustilide, were determined qualitatively within 60 min under the selected HPLC condition. Abbreviations: YGS, Yi-Gan-San; AU, arbitrary perfusion units; 3D, three-dimension; HPLC, high-performance liquid chromatography.

YGS is widely used as a traditional herbal medicine that is composed of seven dried medicinal herbs: *Uncis ramulus*, *Cnidii rhizoma*, *Bupleuri radix*, *Atratylodis Lanceae rhizoma*, Poria, *Angelicae radix* and *Glycyrrhizae radix* in specific ratios. Each plant material was identified by external morphology, and the marker compound of the plant specimen according to the Taiwan Pharmacopoeia standard. In Figure 1, we used 3D high-performance liquid chromatography (HPLC) and UV detection methods to analyze individual active substances and confirm which medicinal material corresponds to the chromatographic peak of YGS. We adopted chlorogenic acid, ferulic acid, liquiritin, glycyrrhizic acid, atractylenolide III and ligustilide, and other standard products to prepare a standard solution, and analyzed and compared the standard and sample solution with the same analytical method. Our 3D HPLC data showed the bioactive substances of YGS were chlorogenic acid, ferulic acid, liquiritin, glycyrrhizin, atractylenolide III, ligustilide, and were determined qualitatively within 60 min. The possible function of these bioactive substances was described in detail separately in the discussion section.

### 2.2. YGS Treatment Shows Better DPPH Free Radical Scavenging Activity

**Figure 2:**
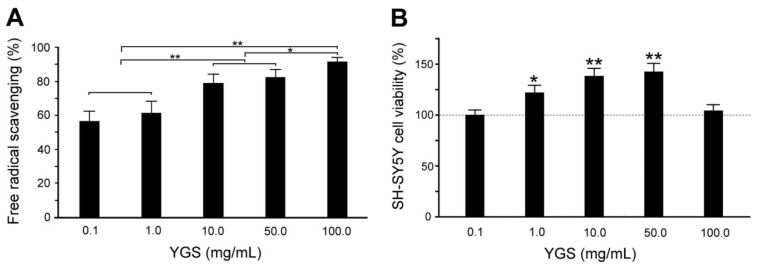
Antioxidant capacity and cytotoxicity of YGS treatment. (**A**) Quantified scavenging activities of free radicals are significantly greater with concentrations of YGS treatments by DPPH assay (*N* = 3 for each group). (**B**) Quantified relative SH-SY5Y cell viability was significantly greater with concentrations of YGS treatments by MTT assay (*N* = 3 for each group). Values are mean ± SEM (** *p* < 0.01, * *p* < 0.05, one-way ANOVA followed by a Student–Newman–Keuls multiple comparisons post-test). Abbreviations: YGS, Yi-Gan-San; AU, DPPH, 1,1-diphenyl-2-picrylhydrazyl; L-AA, L-Ascorbic acid; MTT, 3-(4,5-Dimethylthiazol-2-yl)-2,5-diphenyltetrazolium bromide; SEM, standard error of the mean; ANOVA, analysis of variance.

Figure 2A shows quantified DPPH free radical scavenging activities of YGS extracts at varying concentrations. By using the radical scavenging activity of L-ascorbic acid as the reference standard, we measured 56.4–91.7% of radical scavenging activity under 0.1–100 mg/mL YGS extract treatments. Quantified DPPH radical scavenging activities of YGS extract treatments at 0.1 and 1.0 mg/mL had similar free radical scavenging activity (56.4–61.2%), while there were significant differences (*p* < 0.01, Figure 2A) for those YGS extract treatments at 10–100 mg/mL (78.3–91.7%) from YGS extract treatments at 0.1 and 1.0 mg/mL.

### 2.3. YGS Treatment Shows Higher Cell Viability of SH-SY5Y Cells

Figure 2B shows the quantified cell viability of human neuroblastoma SH-SY5Y cells at different concentrations of YGS extracts treatment. Significant cell viability of SH-SY5Y cells was observed under YGS treatment at 0.5–10 mg/mL. When compared to sham treatment, the cell viability of SH-SY5Y cells was significantly enhanced from 120.9–140.8% under YGS treatment at 0.5–10 mg/mL (*p* < 0.01–0.05, Figure 2B). However, the cell viability of SH-SY5Y cells under YGS treatment at 20 mg/mL has no significant difference from those with sham treatment (*p* > 0.05, Figure 2B). Our results reveal that SH-SY5Y cells show better cell viability only under YGS treatment from 0.5–10 mg/mL.

### 2.4. YGS Treatment Shows Higher Survival Rate for Aβ_42_ Flies

**Figure 3:**
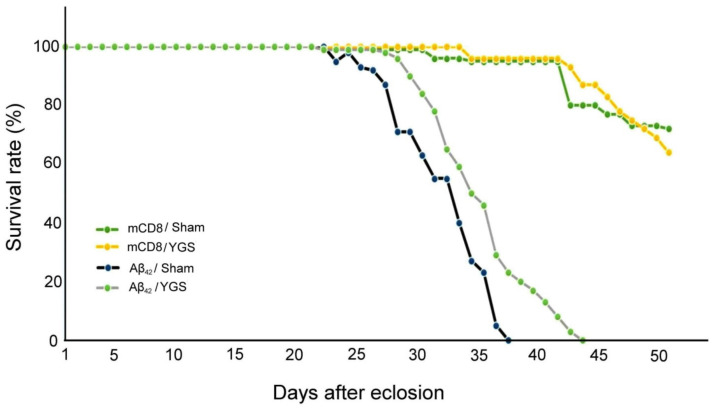
The survival rate of mCD8 and Aβ_42_ flies under sham and YGS treatments. Days after eclosion of mCD8 flies (normal control) with sham (*N* = 300) and YGS treatment (*N* = 300) are longer than those of Aβ_42_ flies with sham (*N* = 300) and YGS treatment (*N* = 300), and days after eclosion of Aβ_42_ flies with YGS treatment are longer than those of Aβ_42_ flies with sham treatment. In other words, YGS treatment should be able to prolong survival duration for Aβ_42_ flies. Abbreviations: YGS, Yi-Gan-San; Aβ, amyloid-beta.

Figure 3 shows the survival rate of mCD8 and Aβ42 flies under sham and YGS treatments. Both days after eclosion of mCD8 flies (as normal control) with sham and YGS treatment are longer than those of Aβ_42_ flies with sham and YGS treatment, and days after eclosion of Aβ_42_ flies with YGS treatment are longer than those of Aβ_42_ flies with sham treatment. Our results reveal that YGS treatment should be able to prolong survival duration for Aβ_42_ flies.

### 2.5. YGS Treatment Shows Better Climbing Index for Aβ_42_ Flies

**Figure 4:**
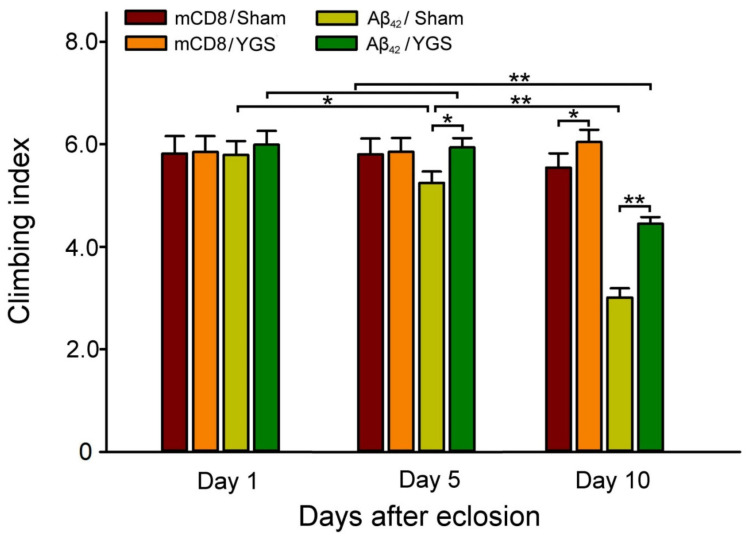
Climbing index of mCD8 and Aβ_42_ flies at 1, 5, 10 days after eclosion under sham and YGS treatments. The quantified climbing index of Aβ_42_ flies at 5 and 10 days after eclosion under YGS treatment was significantly greater than those Aβ_42_ flies under sham treatment (*N* = 30 for each group). In other words, YGS treatment should be able to enhance the climbing index for Aβ_42_ flies. Values are mean ± SEM (** *p* < 0.01, * *p* < 0.05, two-way ANOVA followed by a Student–Newman–Keuls multiple comparisons post-test). Abbreviations: YGS, Yi-Gan-San; Aβ, amyloid-beta; SEM, standard error of the mean; ANOVA, analysis of variance.

Figure 4 shows a quantified climbing index of mCD8 and Aβ42 flies at 1, 5, and 10 days after eclosion under sham and YGS treatments. Our results show a quantified climbing index of Aβ_42_ flies at 5 and 10 days after eclosion, and under YGS treatment was significantly greater than those Aβ_42_ flies under sham treatment (*N* = 30 for each group, *p* < 0.01–0.05, Figure 4). In other words, YGS treatment should be able to enhance the climbing index for Aβ_42_ flies.

### 2.6. YGS Treatment Shows Greater Green Fluorescent Protein (GFP) Fluorescence in the External Eyes for GFP-Aβ_42_ Flies

**Figure 5:**
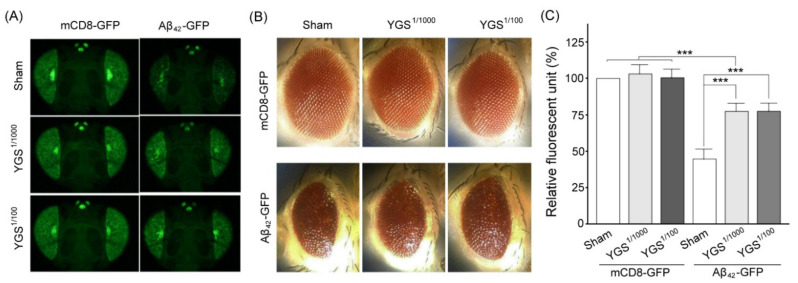
Eye GFP expressions of mCD8-GFP and Aβ_42_-GFP flies under sham and YGS treatments. (**A**) GFP expressions of external eyes in mCD8-GFP (normal control) flies under sham and YGS treatments were obviously greater than those of Aβ_42_-GFP under sham and YGS treatments, while GFP expressions of external eyes in the Aβ_42_-GFP flies under YGS treatment were obviously greater than those of Aβ_42_-GFP flies under sham treatment. (**B**) Completeness of external eyes in mCD8-GFP flies under sham and YGS treatments was obviously better than those of Aβ_42_-GFP under sham and YGS treatments, while completeness of external eyes in the Aβ_42_-GFP flies under YGS treatment was obviously better than those of Aβ_42_-GFP flies under sham treatment. (**C**) Quantified GFP fluorescence of external eyes of mCD8-GFP flies under sham and YGS treatments were significantly greater than those of Aβ_42_-GFP under sham and YGS treatments, while quantified GFP expressions of external eyes in the Aβ_42_-GFP flies under YGS treatment was significantly greater than those of Aβ_42_-GFP flies under sham treatment (*N* = 30 for each group). Values are mean ± SEM (*** *p* < 0.001, two-way ANOVA followed by a Student–Newman–Keuls multiple comparisons post-test). Abbreviations: YGS, Yi-Gan-San; Aβ, amyloid-beta, GFP, green fluorescent protein; SEM, standard error of the mean; ANOVA, analysis of variance.

We modeled GFP fluorescence in Aβ_42_-expressing flies and observed that it was more sensitive and suitable for analyzing Aβ_42_ toxicity. As shown in Figure 5, GFP fluorescence in the external eyes of GFP-Aβ_42_ flies was weaker than those of GFP-WT flies. When GFP-Aβ_42_ flies were treated with 0.1% and 1% YGS treatments, GFP fluorescence in the external eyes was stronger than those of GFP-Aβ_42_ flies with sham treatment (Figure 5A). We quantified and then compared that GFP fluorescence in the external eyes of GFP-mCD8 flies was significantly stronger than those of GFP-Aβ_42_ flies with sham, 0.1% and 1% YGS treatments (*N* = 30 for each group, *p* < 0.01, Figure 5B); while GFP fluorescence in the external eyes of GFP-Aβ_42_ flies with 0.1% and 1% YGS treatments was significantly stronger than those of GFP-Aβ_42_ flies with sham treatment (*N* = 30 for each group, *p* < 0.01, Figure 5B). In addition, GFP fluorescence in the external eyes of GFP-Aβ_42_ flies with 1% YGS treatment was significantly stronger than those of GFP-Aβ_42_ flies with 0.1% YGS treatment (*N* = 30 for each group, *p* < 0.05, Figure 5B). The results revealed that GFP-Aβ_42_ flies showed increasing GFP fluorescence in the external eyes with an increasing dose of YGS treatment.

### 2.7. YGS Treatment Shows Reduced Aβ Expression for Aβ_42_ Flies by IMR Assay

**Figure 6:**
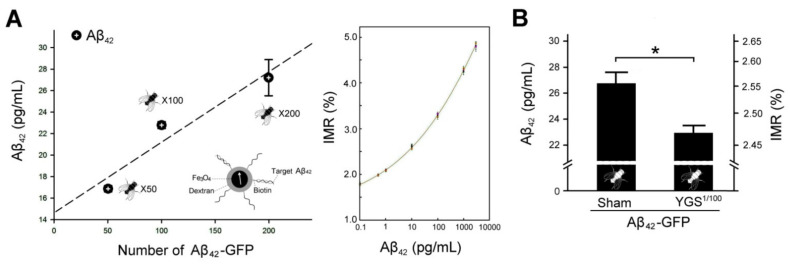
IMR assay of Aβ_42_ expressions for Aβ_42_-GFP flies under sham and YGS treatments. (**A**) Left shows a relationship between Aβ_42_ concentrations and the number of Aβ_42_-GFP flies by IMR assay. A magnetic nanoparticle coated with Aβ_42_ bioprobes was shown. Right shows the IMR value was increased with the Aβ_42_ concentration. (**B**) Quantified Aβ_42_ concentration of Aβ_42_-GFP flies under sham treatment was significantly greater than those of Aβ_42_-GFP flies under YGS treatment (*N* = 100 for each group). Values are mean ± SEM (* *p* < 0.05, one-way ANOVA followed by a Student–Neman–Keuls multiple comparisons post-test). Abbreviations: YGS, Yi-Gan-San; Aβ, amyloid-beta, GFP, green fluorescent protein; IMR, immunomagnetic reduction; SEM, standard error of the mean; ANOVA, analysis of variance.

We used a highly sensitive IMR assay that can detect ultralow concentrations of Aβ protein in human blood for early diagnosis of AD [23,24,25]. We established a curve of the relationship between Aβ_42_ expression and the number of GFP-Aβ_42_ flies by using an IMR assay. Our results showed that the increase in the IMR signal as Aβ_42_ concentration increased was linked to the number of Aβ_42_-expressing flies (Figure 6A, Right). In addition, we plotted a relationship curve and then found that the relationship between IMR signals and Aβ_42_ expression is positively correlated (Figure 6A, Left). IMR signals (in percentages) for Aβ_42_ concentrations between 0.1 and 10,000 pg/mL were used to explore the analytical relationship, which followed a logistic function. According to the relationship curve of Figure 6A, we quantified and then compared that the Aβ_42_ concentration of GFP-Aβ_42_ flies with sham treatment was significantly stronger than those of GFP-Aβ_42_ flies with 1% YGS treatment (sham, 26.4 pg/mL vs. YGS, 22.8 pg/mL, *p* < 0.05, Figure 6B). The results revealed down-regulation of Aβ_42_ expression for GFP-Aβ_42_ flies under YGS treatment.

### 2.8. YGS Treatment Shows Reduced Aβ Expression for GFP-Aβ_42_ Flies by Western Blotting Assay

**Figure 7:**
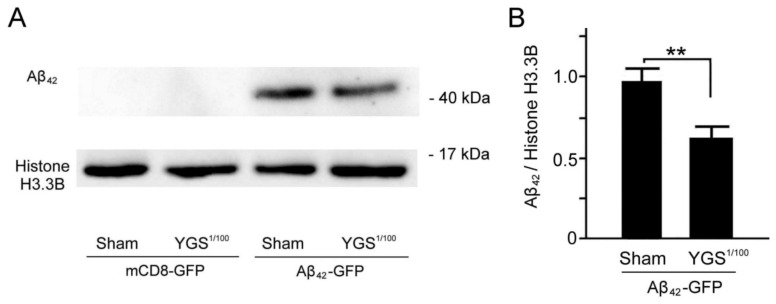
Western blotting analysis of Aβ_42_ expressions of Aβ_42_-GFP flies under sham and YGS treatments. (**A**) An example of Western blotting of Aβ_42_ expressions of Aβ_42_-GFP flies under sham and YGS treatments. (**B**) Quantified Aβ_42_ expressions of the Aβ_42_-GFP flies under YGS treatment were significantly weaker than those of Aβ_42_-GFP flies under sham treatment (*N* = 100 for each group). Values are mean ± SEM (** *p* < 0.01, two-way ANOVA followed by a Student–Newman–Keuls multiple comparisons post-test). Abbreviations: YGS, Yi-Gan-San; Aβ, amyloid-beta, GFP, green fluorescent protein; kDa, kilodaltons; WT, wild-type; SEM, standard error of the mean; ANOVA, analysis of variance.

For further verification, we used Western blotting to examine the Aβ_42_ expressions of mCD8 and Aβ_42_ flies under sham and YGS treatments, shown in Figure 7. Our results showed that Aβ_42_ expressions of mCD8 flies under sham and YGS treatments are quite low, while the Aβ_42_ expression of Aβ_42_ flies under sham treatment was greater than those of GFP-Aβ_42_ flies under YGS treatment (Figure 7A). We quantified and then compared that Aβ_42_ concentrations of Aβ_42_ concentrations of Aβ_42_ flies under sham treatment are significantly greater than those of GFP-Aβ_42_ flies under YGS treatment (sham, 0.96 vs. YGS, 0.61, *p* < 0.01, Figure 7B). The results further confirmed the down-regulation of Aβ_42_ expression for Aβ_42_ flies under YGS treatment.

## 3. Discussion

Oxidative stress has been implicated in the progression of a number of neurodegenerative diseases, including Alzheimer’s disease (AD), Parkinson’s disease (PD) and amyotrophic lateral sclerosis (ALS) [27]. These diseases are characterized by extensive oxidative damage to lipids, proteins and DNA. Oxidative stress is the result of an imbalance in the pro-oxidant/antioxidant homeostasis, leading to a generation of toxic reactive oxygen species (ROS). Aβ can generate H_2_O_2_ with the further generation of ROS through Fenton chemistry. To prove YGS has a neuroprotective function, we first examine whether YGS has the antioxidant ability by using the DPPH assay. In this study, we found that YGS has a good antioxidant ability because YGS extracts show significant DPPH radical scavenging activity at concentrations of 0.1–100 mg/mL. Bioactive markers of YGS were chlorogenic acid, ferulic acid, liquiritin, glycyrrhizin, atractylenolide III, and ligustilide that were determined qualitatively. It has been reported that YGS has neuroprotective effects and rescues neurons, possibly via the PI3K/Akt pathway [28]. Chlorogenic acid, an active compound of Uncaria, could protect against neurodegeneration with supplementation via inhibiting oxidative stress in the brain [29,30,31,32]. In addition, chlorogenic acid treatment can protect Aβ-induced injury in SH-SY5Y neurons and alleviate cognitive impairments in AD transgenic mice via enhancing the activation of the mTOR/TFEB signaling pathway [33]. Ferulic acid, an active compound of Angelica, can delay Aβ-induced pathological symptoms [34]. Liquiritin, an active compound of Licorice, can attenuate rheumatoid arthritis via reducing inflammation, inhibiting the MAPK signal pathway [35], and ameliorating Aβ-induced spatial learning and memory impairment by inhibiting oxidative stress and neural apoptosis [36]. Glycyrrhizin, another active compound of Licorice, can ameliorate inflammatory pain via blockage of the HMGB1-TLR4- NF-kB pathway [37], and prevent cognitive impairment in aged mice by reducing neuroinflammation and AD-related pathology [38]. Atractylenolide III, an active compound of Atractylodes, has anti-inflammatory and neuroprotective effects that may serve as a therapeutic agent in the treatment of depression [39]. Ligustilide, an active compound of Chuanxiong, can improve aging-induced memory deficit by regulating mitochondrial related inflammation and inhibiting oxidative stress in SAMP8 mice [40].

In this study, we modeled GFP fluorescence in Aβ_42_-expressing flies that were more sensitive and suitable for analyzing Aβ_42_ toxicity and identified relevant therapeutic compounds. *Drosophila* Aβ models can help us to approach AD studies in uncovering crucial mechanisms and pathways. In addition, *Drosophila* models can be developed as an excellent tool for various drug testing purposes. As suggested from our results, YGS treatment could significantly reduce GFP fluorescence in the external eyes. These results should provide supporting evidence for the neurotherapy of YGS in an Alzheimer’s disease model of *Drosophila melanogaster* by alleviating Aβ_42_ expression. Although *Drosophila* was an excellent tool for studying AD mechanisms and pathways, there are still risks when using it as a disease model because the pathology may be unique to vertebrates and cannot be transformed into the invertebrate *Drosophila*.

Since the expression of Aβ in the *Drosophila* brain tissue is extremely low, it is very important to find a suitable analytical method to detect the Aβ change. We found that IMR, an ultra-high-sensitivity technology, should be very suitable for detecting ultralow concentrations of Aβ protein in the *Drosophila* brain tissue for diagnosis of AD through the use of antibody-functionalized magnetic nanoparticles dispersed in aqueous solution and the superconducting-quantum-interference-device (SQUID) [23,24,25]. Most studies utilizing SQUID IMR have focused on exploring the relationship between Aβ protein in plasma and in CSF [25,26]. We have achieved promising results in terms of the feasibility of detecting AD in *Drosophila* brain tissue. Our Western blotting data confirmed the results that down-regulation of Aβ_42_ expression for Aβ_42_ flies under YGS treatment by IMR assay is credible. As far as we know, this study should be the first application of IMR technology to detect Aβ protein expressions in the *Drosophila* brain tissue for the diagnosis of AD.

The exact mechanism by which YGS treatment alleviates Aβ_42_ expression remains unclear. Therefore, further clinical trials are warranted to verify the benefits of YGS treatment in AD patients.

## 4. Materials and Methods

### 4.1. Yi-Gan-San (YGS) Preparation

The YGS, an over-the-counter drug called Shun-Ning-Yi, in Taiwan, was manufactured by the Sun-Ten Pharmaceutical company, New Taipei City, Taiwan. The Ministry of Health, Labor and Welfare of Japan has approved this as a remedy for neurosis, insomnia and irritability in children. YGS is a traditional herbal medicine consisting of seven herbs: *Uncis ramulus*, *Cnidii rhizoma*, *Bupleuri radix*, *Atratylodis Lanceae rhizoma*, Poria, *Angelicae radix* and *Glycyrrhizae radix* in specific ratios. As suggested by the Sun-Ten Pharmaceutical company, YGS is a Chinese herbal formula and its mass fraction of constituents was composed of Bupleurum 2 g, Licorice 2 g, Chuanxiong 3.2 g, Angelica 4 g, Atractylodes 4 g, Poria 4 g, and Uncaria 4 g per 100 g of the final product. In Taiwan, YGS was traditionally used as powders. In the prescription of YGS powder, such as *Angelica sinensis* and Chuanxiong, it is rich in volatile oil, which can retain a high content of volatile oil when used in powder, while the volatile oil can be easily volatilized when used in soup. YGS follows the traditional concept of using powder as medicine, mixing crude drug powder according to the proportion of traditional Chinese medicine formula, and then adding a small amount of starch for granulation, with the proportion of starch being 3.3%.

### 4.2. Phytochemical Screening

Before the phytochemical screening, precisely weigh 0.5 g of the YGS powder, ultrasonically shake with 20 mL of 70% methanol at room temperature for 15 min, then shake at 160 rpm for 20 min in a 40 °C water bath, and then centrifuge to take the supernatant. Another 20 mL of 70% methanol was added to the precipitate after centrifugation, ultrasonically shaken for 15 min at room temperature, and then centrifuged again at 160 rpm for 20 min in a 40 °C water bath, and the supernatant was taken. The two supernatants were combined and made up to 50 mL with 70% methanol, mixed evenly, and filtered through a 0.45 µm filter to obtain the test solution. The chromatographic fingerprint analysis of YGS was conducted using 3D HPLC (Burdick & Jackson, Gyeonggi-do, Korea). To confirm which bioactive substances of YGS were in the chromatographic peaks, we used chlorogenic acid, ferulic acid, liquiritin, glycyrrhizic acid, atractylenolide III and ligustilide and other standard products to prepare a standard solution and analyze and compare the standard and sample solution with the same analytical method. HPLC analysis systems regarding column type and identification are described: the model of controller and pump pressurized system was a Waters 600, the degasser was a Waters In-Line Degasser AF, the autosampler was a Waters 717 plus, the photodiode array detector was a Waters 2996, the pre-column was a Lichrospher RP-18 endcapped (5 μm, ID × L = 4.0 × 10 mm, Merck), and the analytical column was a Cosmosil 5C18-MS-II (5 μm, ID × L = 4.6 × 250 mm, Nacalai tesque). HPLC analysis conditions regarding mobile phase, flow rate and detector time are described: column temperature was 35 °C, the flow rate was 1.0 mL/min, and analysis time was 65 min.

### 4.3. DPPH Assay

Antioxidant activities of the YGS treatment were assessed by DPPH assay. YGS extracts that were diluted in distilled water in a concentration range of 0.1 to 100 mg/mL were mixed with 100 μL of 1.5mM/mL DPPH (D9132, Sigma-Aldrich Co., St. Louis, MO, USA) in methanol in a 96-well plate. After 30 min at room temperature, the absorbance of the samples was recorded. The color changes were recorded spectrophotometrically at 517 nm using a microplate spectrophotometer (µQuant™, BioTek Intruments, Inc., Winooski, VT, USA). Appropriate blanks (methanol) and standards (L-ascorbic acid in water, L-AA; A5960, Sigma-Aldrich Co., St. Louis, MO, USA) were recorded simultaneously. Each assay was carried out in triplicate. The DPPH scavenging was calculated by using the following expression:

DPPH scavenging (%) = 100 × [(absorbance of sample + DPPH) − (absorbance of sample blank)]/[(absorbance of DPPH) − (absorbance of methanol)]

Concentrations of YGS that cause 50% scavenging (IC_50_) were calculated from the graph in which scavenging activity was plotted against the corresponding YGS concentration.

### 4.4. MTT Assay under YGS Treatment

Triplicate cultures of 1 × 10^5^ SH-SY5Y cells per well for each 24-well plate. After the YGS or sham treatment, we added 0.5 mg/mL 3-(4,5-dimethylthiazol-2-yl)-2,5-diphenyltetrazolium bromide (MTT, M5655, Sigma-Aldrich Co., St. Louis, MO, USA) to the culture media. SH-SY5Y cells were incubated for 1 h at 37 °C in a humidified atmosphere (95% air and 5% CO_2_), and then the MTT solution was discarded, and 100 μL of dimethyl sulfoxide (DMSO, Sigma-Aldrich Co., St. Louis, MO, USA) was added. The absorbance was read at an optical density (OD) of 570 nm with a microplate spectrophotometer (µQuant™, BioTek Intruments, Inc., Winooski, VT, USA).

### 4.5. Animals and Experimental Design

Aβ_42_-expressing flies were generated by the Goldstein Laboratory (stock No. 32038). In this study, Aβ_42_-expressing flies (Aβ_42_-GFP flies) and their WT (mCD8-GFP flies) were used to examine the effect of YGS treatment by comparing retinal GFP expression without the need for histological assessment. The stronger Aβ_42_ expressions in GFP-Aβ_42_ flies and the weaker retinal GFP expression was monitored [15]. In this study, unless otherwise specified, both GFP-Aβ_42_ and their WT flies were cultured and maintained on a standard cornmeal-yeast-agar medium at 25 °C and 60% humidity, in which the standard medium of GFP-Aβ_42_ was uniformly mixed with 1% and 0.1% YGS by weight of the medium.

### 4.6. Survival Rate and Behavior Analysis in Drosophila

Aβ_42_ flies and mCD8 flies (*N* = 300 for each group) were exposed to sham and 1.0% YGS treatment. The flies were observed daily for the incidence of mortality, and the survival rate was determined by counting the number of dead flies for 50 days. The data were subsequently analyzed and plotted as cumulative mortality and percentage survival after the treatment period.

The negative geotaxis assay was used to evaluate the locomotor performance of flies (*N* = 30 for each group). In brief, after the treatment period of 5 days, the flies from each group were briefly immobilized in ice and transferred into a clean tube (11 cm in length, 3.5 cm in diameter) and labeled accordingly. The flies were initially allowed to recover from immobilization for 10 min and thereafter were tapped at the bottom of the tubes. Observations were made for the total number of flies that crossed the 6 cm line within a period of 6 s and recorded. The results were expressed as a percentage of flies that escaped beyond a minimum distance of 6 cm in 6 s during three independent experiments.

### 4.7. Retinal GFP Expression Assay in Drosophila

We took an image of the outer Drosophila eye with an Olympus-BH2 microscope. Tissue sections and fluorescence were imaged using a Leica DM IRB fluorescence microscope. For examination with fluorescence microscopy, we removed the Drosophila head with spring scissors between the head and the thorax that was arranged in pairs so that individual experimental flies could be imaged and compared directly to individual flies from their respective control groups. Fluorescence was quantified by using publicly available NIH Image J software. The mean retinal fluorescence from Aβ_42_ flies was normalized to the mean fluorescence from mCD8 flies (30 files for each group).

### 4.8. IMR Assay in Drosophila

*Drosophila* (100 files for each group) was homogenized and mixed with a reagent, which consisted of magnetic nanoparticles that were functionalized with monoclonal antibodies against a target protein, and then dispersed in PBS of pH 7.2 (MagQu Co., Ltd.) at room temperature. The magnetic nanoparticles used were dextran-coated Fe_3_O_4_ particles (MF-DEX-0060, MagQu Co., Ltd.). For each sample at each target protein concentration, IMR signal measurements were performed in duplicate. The signals were converted to biomarker concentrations using standard curves. All plasma samples were blinded for IMR measurements. The tau reagent (MF-TAU-0060, MagQu Co., Ltd.) contained magnetic nanoparticles immobilized with a monoclonal antibody (T9450, Sigma) against human tau protein. The Aβ_1–42_ reagent (MF-AB2-0060, MagQu Co., Ltd.) contained magnetic nanoparticles coated with a monoclonal antibody against human Aβ_1–42_ protein. These reagents were superparamagnetic, with a saturated magnetization of 0.3 emu/g. A SQUID-based AC magnetic susceptometer (XacPro-S, MagQu Co., Ltd.) was used to determine the time-dependent AC magnetic susceptibility, which approximates the association between magnetic nanoparticles and target protein molecules in the plasma [24] of each mixture. The IMR signal, which refers to the reduction in magnetic susceptibility caused by the association between magnetic nanoparticles and the target protein molecule, as detected by the magnetic susceptometer, represents the concentration of the target protein.

### 4.9. Western Blotting in Drosophila

Total proteins were extracted from the head tissue of *Drosophila* following the treatment described (100 files for each group). The removed tissue was homogenized in a buffer solution that was placed on ice for one hour and then centrifuged at 4 °C for 13,000 rpm for another 20 min. The separated solution was quantified by using a BCA protein assay kit (Thermo Fisher Scientific Inc. Waltham, MA, USA). Proteins were separated on 12.5% or 15% SDS polyacrylamide gels (Bionovas Pharmaceuticals Inc., Washington, DC, USA), and proteins were transferred to polyvinylidene difluoride membranes (GE Healthcare Life Sciences, Barrington, IL, USA). The antibodies used in this study were anti-Histone H3.3B (Thermo Fisher Scientific Inc.), and anti-amyloid-beta (anti-Aβ) (Covance Cat#SIG-39220, BioLegend, Dedham, MA, USA). Antibodies were detected by suitable horseradish peroxidase (HRP)-conjugated secondary antibody (Santa Cruz Biotechnology Inc.), and then proteins’ immunoreactive bands were visualized by the enhanced chemiluminescence (ECL) substrate (Millipore, Billerica, MA, USA), and the band intensities were quantified with the Image J analysis software (version 1.48t, Wayne Rasnabd, USA).

### 4.10. Statistical Analysis

All data are presented as means ± standard errors of the mean. One-way or two-way analysis of variance was performed, followed by the Student–Newman–Keuls post hoc test. The *p*-values of at least < 0.05 were considered significant. All data are obtained in at least three independent experiments.

## 5. Conclusions

Transgenic Drosophila Aβ models have successfully provided valuable information for studying AD mechanisms and pathways. In addition, Drosophila provided valuable drug testing in vivo experiments for YGS treatment. For in vitro experiments, our results showed that YGS treatment has a good antioxidant ability and low cytotoxicity. For in vivo experiments, our results showed that YGS treatment can reduce Aβ and Tau expressions in Drosophila melanogaster by IMR assay and Western blotting that were quite consistent with the change in appearance traits. To the best of our knowledge, this study was the first to conduct an evidence-based investigation of the effectiveness of alternative therapy with the traditional Chinese medicine YGS treatment in alleviating Aβ neurotoxicity of *Drosophila melanogaster* assessed by highly sensitive IMR assay and evaluated from appearance traits in GFP expression of external eyes.

## Data Availability

The data is confidential.
